# A Brain for Speech. Evolutionary Continuity in Primate and Human Auditory-Vocal Processing

**DOI:** 10.3389/fnins.2018.00174

**Published:** 2018-03-27

**Authors:** Francisco Aboitiz

**Affiliations:** Centro Interdisciplinario de Neurociencias, Escuela de Medicina, Pontificia Universidad Católica de Chile, Santiago, Chile

**Keywords:** speech, working memory, evolution, animal vocalization, arcuate fasciculus

## Abstract

In this review article, I propose a continuous evolution from the auditory-vocal apparatus and its mechanisms of neural control in non-human primates, to the peripheral organs and the neural control of human speech. Although there is an overall conservatism both in peripheral systems and in central neural circuits, a few changes were critical for the expansion of vocal plasticity and the elaboration of proto-speech in early humans. Two of the most relevant changes were the acquisition of direct cortical control of the vocal fold musculature and the consolidation of an auditory-vocal articulatory circuit, encompassing auditory areas in the temporoparietal junction and prefrontal and motor areas in the frontal cortex. This articulatory loop, also referred to as the phonological loop, enhanced vocal working memory capacity, enabling early humans to learn increasingly complex utterances. The auditory-vocal circuit became progressively coupled to multimodal systems conveying information about objects and events, which gradually led to the acquisition of modern speech. Gestural communication accompanies the development of vocal communication since very early in human evolution, and although both systems co-evolved tightly in the beginning, at some point speech became the main channel of communication.

## Introduction

*Homo sapiens* is an outstanding and successful species, arguably due to our capacity for speech and language. In previous works, my colleagues and I have emphasized that the emergence of the phonological loop, an auditory-vocal circuit involved in verbal working memory, was a radical innovation in speech origins, as it expanded auditory-vocal short term memory capacity, enabling early humans to learn increasingly complex vocal utterances (Aboitiz, 1995, 2012, 2017; Aboitiz and García, 1997; Aboitiz et al., 2006a,b, 2010). In this article, I will review and extend these ideas, some but not all of which have been put forward recently (Aboitiz, 2017). Basically, the main contribution of this paper is to provide a comprehensive but summarized scenario, starting from the preconditions existent in non-human primates and the subsequent development of a sophisticated neural control of vocalizations in early humans.

## Preconditions to speech: primate adaptations

Humans belong to the order Primates, which originated in the late Cretaceous, some 65 million years ago. Primates are characterized by arboreal habits, superior grasping abilities and good frontal vision, initially associated with nocturnal habits. More derived primates are diurnal animals, and display a complex visual system with color vision, which is useful for fruit recognition (Fleagle, 2013).

### Primate brains

Another feature of primates is their brain size, that has been related by many to higher cognitive capacity. Not only primates have a brain that doubles the size of other mammals of the same body size, but also they display a much higher neuronal density than that of other mammals in their cerebral cortices (Herculano-Houzel et al., 2015). Humans have the largest brain and the highest number of neurons of all primates (Herculano-Houzel et al., 2015). This increase in brain size and neuron number has gradually developed along the *Homo* lineage, partly associated with increase in body size but growing disproportionately to the latter, making our brains the largest in size (and with more neurons) in relation to body size of all animals (Aboitiz, 2017). A contentious issue is whether the prefrontal cortex has grown disproportionately in primates, especially in humans. Altogether, the recent evidence suggests that in humans and primates, the prefrontal cortex grows concomitantly with other higher order areas in the parietal and temporal regions, while lower order sensorimotor areas evolve more conservatively (see next section) (Margulies et al., 2016).

### Paleoanthropological evidence of human brain evolution

The study of fossil endocasts of hominin brains has provided important information about the size and shape of the brains, which increased in size from some 500 cc. in australopithecines to more than 1,000 cc. in late *Homo erectus*. More modern *Homo* species like Neanderthals, Denisovans, and modern humans show a further increase in brain size up to about 1,500 cc. A contentious issue has been the identification of Broca's language region in early hominin endocasts. Australopithecines lack a human-like Broca's cap region, but specimen KNM-ER 1470 (*H. Rudolfiensis*) displays a more advanced morphology in this area (Holloway, 2017). Compared to other human fossils, Neanderthals and modern humans display an increased depth of the anterior fossa that corresponds to part of Broca's region and relatively wider frontal lobes (Bruner and Holloway, 2010). These are also the only human species with the frontal lobes located entirely over the orbits (Bruner et al., 2014), but the functional implications of these findings are unclear (Balzeau et al., 2014; Bruner, 2017). On the other hand, both humans and apes display larger frontal lobes on the right hemisphere and a larger occipital lobe on the left hemisphere, although asymmetries are more marked in fossil hominins (Bruner, 2017; Holloway, 2017).

The parietal surface of the endocranium has evidenced more clear differences among early humans. Firstly, the lunate sulcus that separates parietal cortex from the primary visual cortex in apes, is absent or very fragmented in modern humans, presumably via expansion of the parietal lobe (Holloway, 2017). Neanderthals and modern humans exhibit wider upper parietal regions than other hominids, and modern humans have these regions larger than Neanderthals (Bruner et al., 2011). The two regions showing most cranial differences are the midsagittal precuneus and the intraparietal sulcus, although both are highly variable even among modern humans (Pereira-Pedro et al., 2017). The precuneus and the intraparietal lobe are important nodes for large scale neural networks including the default mode network and circuits for hand and eye coordination (Bruner, 2017). This evidence fits the increasingly globular shape of the modern human cranium (Neubauer et al., 2018). The expansion of these regions may also relate to increasing hand control and tool making (Stout and Hecht, 2017), and to other functions like orientation, attention, self-awareness, and some aspects of language (see below).

### Hand control and gestures

Primate hands (and feet) are more prehensile than those of other mammals, featuring an opposable thumb suitable for grasping branches and leaves or fruit, that can be brought to the mouth for consumption. Furthermore, their fingers have nails instead of claws, and highly sensitive finger buds below the nails. These morphological features are related to a direct, monosynaptic corticospinal innervation of the cervical spinal cord motoneurons controlling the hand muscles, a character associated with hand dexterity and found only in primates (Fleagle, 2013). Nonetheless, other mammals like rodents have a transient, direct corticospinal projection to hand motoneurons, that is present postnatally but is eliminated during development, a process mediated by the gene PlexA1 (Gu et al., 2017). Importantly, PlexA1 mutant mice maintain the direct corticospinal projection in adulthood, and display enhanced manual dexterity than normal animals. In addition, grasping behavior development requires a neonatal transient visual pathway in primates (Mundinano et al., 2018).

Grasping behavior also depends on complex neural networks involving parietal and premotor areas as critical nodes in a widespread network that includes temporoparietal and prefrontal areas. In this circuit, visual information about both the nature and position of the object to be grasped are used for coordinating a precise motor sequence that includes reaching the object and then grasping it (Borra et al., 2017). A great deal of excitement was produced by the discovery of grasping mirror neurons in area F5 of the ventral premotor cortex of the monkey, which fire both when the monkey executes a grasping action and when it observes another individual performing the action (di Pellegrino et al., 1992). These neurons were soon interpreted as involving a motoric representation on the other's behavior, and were considered as essential to understand the goals and intentions of others by activating one's own motor programs emulating the behavior (Rizzolatti et al., 1996). Afterwards, Rizzolatti and Arbib (1998; Arbib, 2012) put forward the hypothesis that grasping mirror neurons were essential for the origin of language, and revived the theory that the earliest forms of symbolic communication were gestural instead of vocal.

The notion of mirror neurons as representing other agent's intentions or goals has been questioned by some authors and this is now a matter of intense debate (Cook et al., 2014; Hickok, 2014). Concerning the gestural theory of language origins, the core proposal of the present paper is how speech itself was acquired, regardless on whether the first symbols were hand- or mouth- based. Nonetheless, although not an implausible hypothesis, the gestural theory is highly speculative and contestable (Bosman et al., 2005; Aboitiz, 2013). One of its central assumptions is that because monkeys and apes have voluntary control of hands but not of voice, language must have started from manual gestures and was only later transmitted to the vocal system by some unknown mechanism (the theory says very little about speech origins). However, voluntary hand control is widespread among primates and language is uniquely human. Thus, something else than hand control is needed to account for human language. Moreover, monkeys and apes have voluntary control of the lips, which are essential for speech, and orangutans have been shown to imitate human speech (Lameira et al., 2014). In this line, some adherents to the mirror neuron hypothesis propose a role of lip movements and hand-mouth interactions in early human communication (Coudé and Ferrari, in press), but this is disputed by some other mirror neuron theorists (Arbib, 2012). More generally, the conjecture that hand signing made possible the development of vocal plasticity leading to speech contrasts with abundant comparative evidence that voluntary control of vocalizations and vocal learning can evolve without necessity of a hand-grasping circuitry, as it occurs in songbirds, bats and marine mammals (Aboitiz, 2012, 2017). Perhaps more parsimonious is the notion that the human voice developed in parallel and coevolved with hand control.

### Tool making behavior

Tool making behavior is observed in monkeys and apes, but fossil hominids excelled by far the other primate species. In modern humans, stone tool making relies on a network encompassing visual areas, the inferior parietal lobe and ventral premotor areas. Furthermore, the ventral aspect of the superior longitudinal fasciculus (SLF) connects inferior parietal and premotor areas, and is larger and more asymmetric (with the right side larger) in humans than apes (Budisavljevic et al., 2015; Putt et al., 2017; Stout and Hecht, 2017). As these networks may show some overlap with the speech networks to be described below, it is tempting to hypothesize that speech and tool making reinforced each other in human evolution. However, the relation between tool-making behavior and speech is unlikely to be direct, as there is conflicting evidence as to whether spoken instructions improve tool-making learning in modern humans (Putt et al., 2014; Morgan et al., 2015; Cataldo et al., 2018). On the other hand, speech acquisition in children obviously does not depend on tool making behavior. While gesturing and especially imitation were probably more relevant for tool-making behavior in our ancestors, learned vocalizations may have developed as a parallel acquisition associated with social rather than technological demands, in an increasingly complex protoculture where both gestures and vocalizations were essential components of communication (Cataldo et al., 2018). Finally, tool-making requires a clear division of labor between both hands, which may have contributed to the generation of language asymmetries in humans (Uomini and Meyer, 2013; Hecht et al., 2015), although communication constraints may have also been important (see below).

### Vocal and orofacial behavior in non-human primates

Basal primates like lemurs show a strepshirhine condition shared with other mammals, where the lips elevate to the nose. On the other hand, some prosimians like the tarsius and the rest of the primates display a haplorhine condition in which the upper lip becomes separated from the nose by a band of skin, making a continuous lip around the mouth that is used for feeding and communication (Fleagle, 2013). In fact, lips are highly movable in higher primates, and they display a series of social signals using lip movements. Lip-smacking is a common affiliative behavior used by many primates, but there are other types of voiceless calls, like “clicks,” “kisses,” and “whistles,” that are produced by the upper vocal tract, particularly the lips. In fact, non-human primates have a sophisticated, very likely voluntary, neural control of their lips, of which we know little about yet (Lameira et al., 2014; Coudé and Ferrari, in press). Recent reports have described interesting similarities between monkey lipsmacking and human lip movements while speaking, which follow similar developmental trends (Ghazanfar et al., 2012; Morrill et al., 2012). A second organ involved in human speech is the tongue, but more research is needed on how non-human primates use it for feeding or communicating.

Non-human primates are highly vocal animals, that communicate intensely through coordinated calls generated by movements of the laryngeal vocal folds (Belyk and Brown, 2017). Non-human primate vocalizations are usually fixed in structure and species-specific, but can be modulated according to social context, and there is voluntary control of when and what to vocalize (Hage et al., 2013; Hage and Nieder, 2016). Like humans, apes are able to modulate the fundamental frequency of their vocalizations, depending on the listener and social context (Pisanski et al., 2016). Furthermore, in some primates like marmosets, vocalizations develop in infants form a variable structure that gradually consolidates in clustered acoustical signals during maturation, just like in infants and songbirds, a process driven by maternal feedback (Takahashi et al., 2015; Hage et al., 2016). In addition, some primates like gibbons and marmosets engage in reciprocal “conversations” that can last for a long time (Geissmann, 2002; Takahashi et al., 2013). While the gibbon's duets are rather stereotyped in structure, marmosets appear to have some variability in their vocalizations (Thinh et al., 2011; Koda et al., 2013; Hage et al., 2016; Takahashi et al., 2016; Pomberger et al., 2018).

Lieberman (1968) observed that the larynx is in a lower position in the vocal tract in humans than in other primates, which was attributed to the development of a resonance cavity in the upper vocal tract for the production of vowels. More recent studies have found that this character is also present in other animals like male deer, a result of sexual selection for generating lower frequencies and give the impression of a larged body size (Fitch and Reby, 2001). Yet, early humans may have taken advantage of this condition to optimize vowel production. As will be discussed below, only humans among primates have direct cortical control over laryngeal musculature, which may have evolved together with the descent of the larynx in our ancestors.

Nonetheless, a recent study showed that all movements used by humans when speaking can be executed by monkeys, and computer simulations of monkey vocalizations were able to generate human-like speech (Fitch et al., 2016; but see Fitch et al., 2017; Lieberman, 2017). Another study showed that monkeys naturally emit sounds similar to human vowels, but they do not organize them into complex phonological sequences, presumably because they lack direct cortical control of these muscles (Boë et al., 2017). Another aspect of interest is the coordination of lips and larynx during communication. While in most primates, upper vocal tract movements (lips) dissociate from vocalizations (emitted by the lower vocal tract, i.e., the larynx), in human speech these become tightly coordinated. An intermediate situation is found in the “wobble” call of the gelada, in which vocalizations are synchronized with lip smacking (Ghazanfar and Takahashi, 2014a,b). Other interesting findings are the reports of human voice imitation in orangutans, who in addition have incredibly movable lips (Lameira et al., 2016).

### Descending control of face and throat

Vocalizations and orofacial movements are controlled by several brainstem nuclei, such as the trigeminal motor nucleus innervating jaw musculature, the hypoglossal nucleus driving tongue movements, the facial nucleus controlling face and lip movements, and finally the ambiguus nucleus innervating the vocal folds in the larynx. In addition, vocalizations depend on a tight control of respiratory muscles. These nuclei relate to brainstem central pattern generators that produce cyclic activity for behaviors like chewing, swallowing, drinking, laughing and swallowing (Jürgens, 2009; Hage and Nieder, 2016). It is most likely that these circuits were recruited and remodeled for the development of human speech, as for example, respiratory movements have to be much more controlled during speech than during primate vocalizations (Ghazanfar and Rendall, 2008; Ghazanfar and Takahashi, 2014a; Belyk and Brown, 2017).

**Figure 1:**
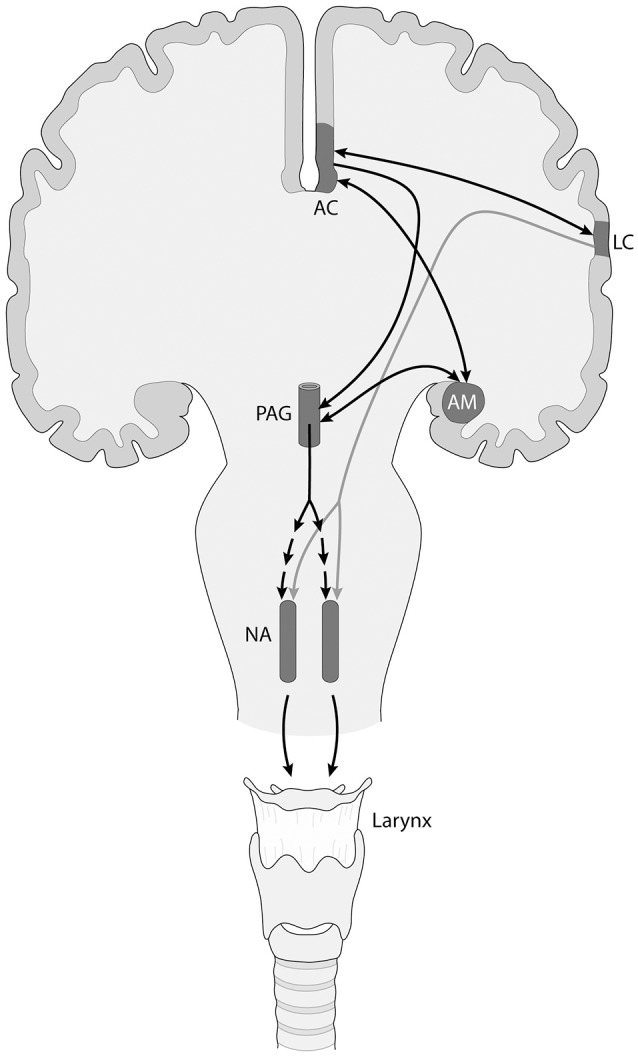
Simplified scheme of the descending neuronal control of the nucleus ambiguus (NA), that controls vocal fold musculature, in primates including humans. There are two different neural networks involved, an emotionally controlled, non-volitional one (black arrows) that includes limbic and other components like the anterior cingulate (AC) cortex and the amygdalar complex (AM), which project to the mesencephalic periaqueductal gray (PAG). In turn, the PAG sends a polisynaptic projection to the neurons of the NA (segmented arrows). In addition, there is a descending projection from the laryngeal motor cortex (LC) to the NA (gray arrow), that exerts voluntary control over vocalizations. These two pathways are connected via the frontal aslant tract (arrow connecting AC with LC). A similar organization is found in the networks controlling the brainstem nuclei innervating the musculature of the upper vocal tract (lips and tongue), which for simplicity are not shown.

In turn, these brainstem circuits are controlled by an upper level network that involves the cingulate cortex, the orbitofrontal cortex, the insula, and the amygdala, which connect to the mesencephalic periaqueductal gray and then reach the pacemaker circuits in the brainstem reticular formation (Figure 1; Simonyan and Jürgens, 2003; Jürgens, 2009; Hage and Nieder, 2016; Holstege and Subramanian, 2016; Coudé and Ferrari, in press). This circuit is considered to be responsible for triggering reflex, non-volitional vocalizations, and is also involved in the rewarding and emotional dimension of communication. In addition to this circuit, but well connected to it, there is a second circuit centered in the motor and premotor orofacial and laryngeal cortices, that is connected with the basal ganglia, thalamus and cerebellum, and is involved in volitional control of vocalizations. While in non-human primates, the laryngeal motor representation is located in the ventral premotor cortex, in humans it is located in the motor cortex, adjacent to the orofacial motor representation (Belyk and Brown, 2017). The human laryngeal motor cortex also participates in respiratory control, and is proposed to be duplicated, with ventral and dorsal components (Belyk and Brown, 2017).

**Figure 2:**
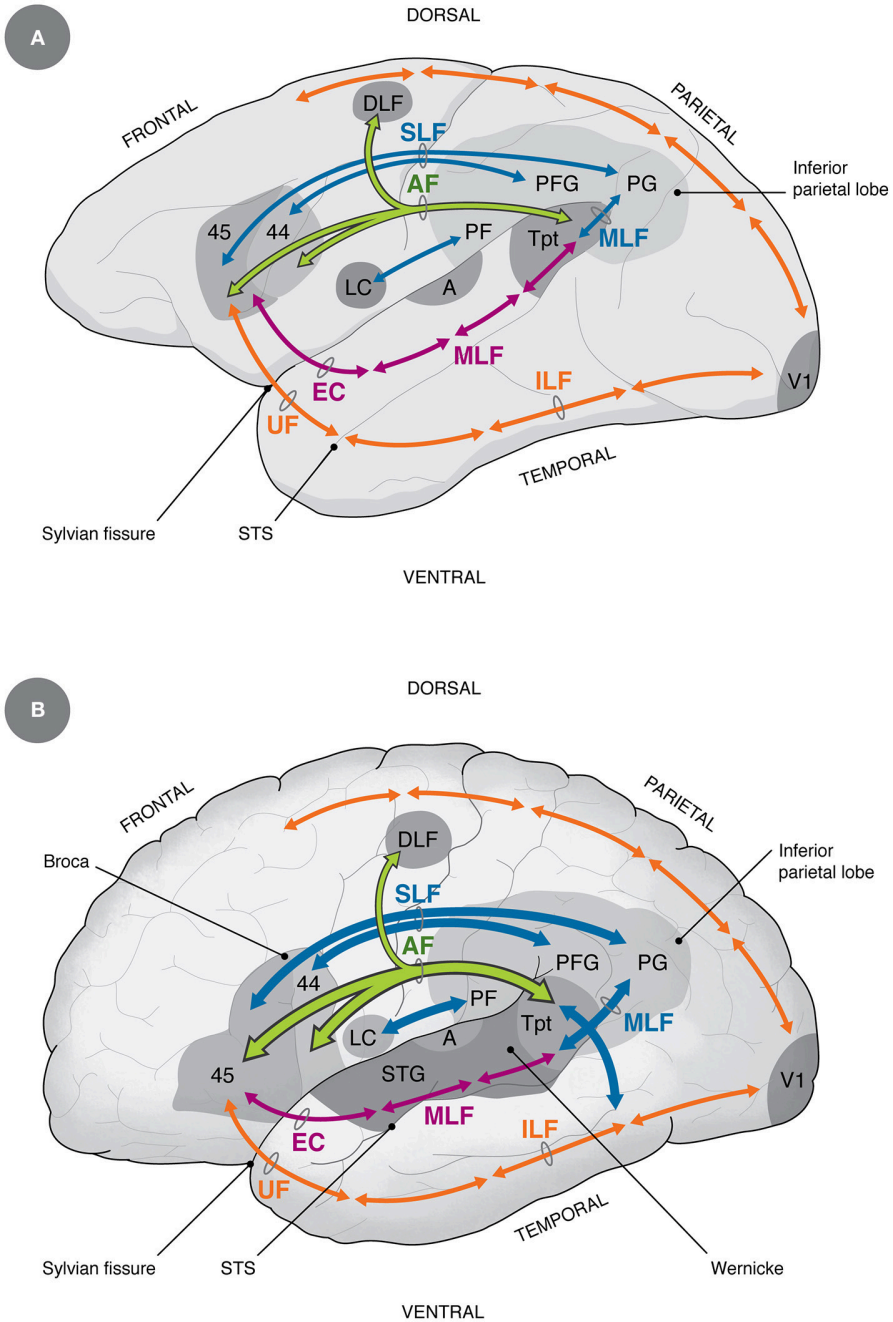
Homology and differences in auditory-vocal cortical connectivity between non-human primates **(A)** and humans **(B)** (Petrides, 2014). The main differences between humans and non-human primates discussed in this paper refer to the increase in size of the AF, the ventral SLF and the posterior MLF (Rilling et al., 2008; Catani and Bambini, 2014; Stout and Hecht, 2017), the increase in connectivity between LC and inferior parietal areas (Kumar et al., 2016), the projection from the dorsal pathway into the medial temporal gyrus (additional blue arrow in humans), which is considered by some as part of the AF (Rilling et al., 2008; Catani and Bambini, 2014), and by others as part of the MLF (Petrides, 2014). Additional differences, not shown in the diagram, are that in humans there is a direct descending control of laryngeal motoneurons (Jürgens, 2009) and increased control of respiratory muscles (Belyk and Brown, 2017). A, primary auditory area; AF, arcuate fasciculus (green); DLF, dorsolateral frontal cortex; EC, extreme capsule; ILF, inferior longitudinal fasciculus (orange); LC, laryngeal and orofacial cortex; MLF, medial longitudinal fasciculus (magenta and blue); PF, PFG, PG, inferior parietal areas; SLF, ventral superior longitudinal fasciculus (blue); STG, superior temporal gyrus; Tpt, cytoarchitectonic area Tpt; UF, uncinate fasciculus; V1, primary visual area. For reference, dorsal and ventral visual pathways are shown in orange.

As mentioned, the non-volitional and the volitional vocalization circuits are interconnected, but their connectivity has been claimed to increase in the human lineage. In this context, the frontal aslant tract connects dorsomedial frontal cortex with ventrolateral frontal and prefrontal cortex, and its maturation has been related to speech acquisition in infants (Catani et al., 2013), which makes it a prime candidate to bridge both circuits (Figure 1). Furthermore, the laryngeal and probably the orofacial motor cortex have connections with somatosensory, inferior parietal and posterior superior temporal (auditory) areas, possibly participating in an audio-vocal circuit that transforms auditory input in vocal output signals and vice versa (Figure 2; Kumar et al., 2016; Hickok, 2017).

The orofacial and laryngeal motor cortices send descending projections to the reticular formation, controlling the distinct cranial motor nuclei. It has been proposed that, as opposed to the rest of primates, in humans the laryngeal cortex sends a direct projection to the nucleus ambiguus controlling the vocal folds (Figure 1), while in other primates these axons reach nearby interneurons that themselves project to the nucleus ambiguus (Jürgens, 2009). A direct projection to the nucleus retroambiguus, controlling respiratory movements has also been proposed (Belyk and Brown, 2017). These characters have been considered to be key for the acquisition of vocal learning capacity in humans. A striking parallelism has been found in songbirds, where there is a direct descending projection from a telencephalic motor nucleus to the cranial nucleus controlling syrinx musculature. Vocal non-learning birds, like non-human primates, lack this direct projection (Petkov and Jarvis, 2012).

### Premotor and prefrontal control of vocal and orofacial behavior

In non-human primates, there is also prefrontal control of the orofacial and laryngeal musculature. Petrides and collaborators reported that stimulation of area 44 in the ventrolateral prefrontal cortex of monkeys (homologous to posterior Broca's area in the human) triggers orofacial movements and very rarely hand movements (Petrides et al., 2005). Coudé et al. (2011) found neurons firing with voluntary vocalizations in the macaque ventral premotor cortex, and Hage and Nieder (2013) reported similar properties in the monkey prefrontal cortex, specifically in area 44 and surrounding regions. Furthermore, neuronal activity in the prefrontal cortex of marmosets has been found to correlate, and even predict, whether an animal will engage or not in a reciprocal, “conversational” loop with another individual (Nummela et al., 2017).

Additional studies have reported neurons with mirror properties for mouth movements in the ventral premotor cortex of the monkey, that activate both during food ingestion and during communication behaviors like lip-smacking (Ferrari et al., 2003, 2017). Like grasping mirror neurons, mouth mirror neurons fire both during the execution and the observation of mouth movements. Interestingly, the mouth representation overlaps with the hand representation in the ventral premotor cortex, where neurons involved in hand, mouth and gestural behavior are intermingled (Coudé and Ferrari, in press). This overlap may be important for hand-mouth coordination behavior, a character that is probably ancestral to mammals but acquires more relevance in primates, both for feeding and communicative purposes (Coudé and Ferrari, in press). Like the laryngeal motor cortex, the representation of face and lips is connected with the non-volitional/emotional vocalization circuit described above, including the anterior cingulate cortex, orbitofrontal cortex, insula, amygdala and other regions (Hage and Nieder, 2016; Ferrari et al., 2017). Acoustical, instead of visual, mirror neuron activity has been also found with sounds that are associated with actions like tearing a paper (Kohler et al., 2002), but to date no visual or acoustical mirror activity has been reported for monkey vocalizations (but see below; Hage and Nieder, 2015).

### Auditory networks in the monkey

The primate auditory cortex is organized in three concentrical rings located in the superior temporal lobe, in which there is a core region containing primary and secondary auditory areas, a belt region surrounding it, that houses higher order auditory regions, and a parabelt area that projects to surrounding cortices of the temporal, parietal and frontal lobes (Kaas and Hackett, 1999). From these regions, two main processing streams emerge: Firstly, a dorsal component projects to inferior parietal and frontal areas, partly emerging from area Tpt, an important node in posterior auditory cortex. Secondly, there is a ventral component that runs anteriorly along the superior temporal lobe, reaching ventrolateral prefrontal areas (Figure 2A). The dorsal component performs time-dependent analyses of the stimulus and is involved in sound localization, while the ventral pathway is related to stimulus identification and has strong connectivity with the limbic, anterior temporal regions (Kaas and Hackett, 1999; Romanski, 2007; Rauschecker, 2012).

The dorsal pathway has been usually associated to the arcuate fasciculus (AF), but there are author differences in the definition of this tract (Catani et al., 2005; Rilling et al., 2008; Petrides, 2014). In this article, I will rely on Petrides' definition of the AF as “those monosynaptic axons that arch around the end of end of the lateral (Sylvian) fissure to link temporo-parietal cortex with frontal cortex” (Petrides, 2014 p. 163; see Figure 2A). Hodological studies in the monkey revealed three main components of this tract: one originating in the ventral superior temporal gyrus (STG) and the upper bank of the superior temporal sulcus (STS) that terminates in prefrontal area 44; another originating in the ventrocaudal STG, the adjacent STS and part of the medial temporal gyrus (MTG) that terminates in area 45; and a third branch originating from the dorsal STG that terminates in dorsolateral frontal cortex, the latter involved in auditory-related eye movements (for review see Petrides, 2014). Additional auditory-related connections have been described between posterior auditory regions and the ventral premotor cortex (Kumar et al., 2016), and between inferior parietal areas and the ventrolateral prefrontal cortex (Petrides, 2014), which will be discussed below.

The subdivision into dorsal and ventral processing streams emulates the well-known organization of the visual system, containing a dorsal spatial-movement pathway that serves to coordinate actions along the superior parietal and frontal lobes, and a ventral pathway along the inferior temporal lobe and ventral-dorsolateral prefrontal cortex involved in visual identification of objects and faces (Figure 2; Goldman-Rakic, 1990, 1995). Interestingly, the ventral visual pathway, traveling along the inferior temporal lobe, projects to areas 47 and 45, partly overlapping with the termination of the auditory ventral pathway, and serving as a link between face and vocal perception (see below; Romanski, 2007).

In areas 12 and 45 of the monkey ventrolateral prefrontal cortex, single auditory neurons have been reported to respond to conspecific vocalizations, which are interspeded with visual neurons responding to conspecific faces (Romanski and Goldman-Rakic, 2002). Furthermore, some single neurons have been found to respond to both kinds of stimuli, and in some cases, these neurons suppress their activity when presented with an incongruous face-voice pair (Sugihara et al., 2006; Diehl and Romanski, 2014). Other studies have observed activity modulation of these neurons by both the emission and the perception of vocalizations, which is reminiscent of mirror neuron activity (Hage and Nieder, 2015). A different line of research has reported that perisylvian regions, including posterior parietal and ventrolateral prefrontal regions, activate during learning of simple artificial grammars and tasks similar to non-word sequencing tasks for humans (Milne et al., 2017; Wilson et al., 2017). These circuits overlap with those involved in syntactic processing in humans, suggesting that ordering and hierarchical processing of human speech and language partly derives from some domain-general mechanism for ordering actions.

## The speech network in humans

The neural substrate for human speech has been analyzed since the times of Paul Broca and Karl Wernicke, who recognized two main speech-related cortical areas, an anterior one in the ventrolateral prefrontal cortex involved in speech production (Broca's area), and a posterior one in the posterior superior temporal lobe involved in speech perception (Wernicke's area). The AF has been classically considered to connect these areas, translating auditory representations into vocal articulatory patterns. This basic concept has been deeply revised in the last years, by virtue of evidence emerging from brain imaging studies depicting a complex network connecting several speech associated regions. In addition, Broca's and Wernicke's areas have been found to be less well defined anatomically than originally thought, and several surrounding regions may contribute to speech comprehension and execution (Fuertinger et al., 2015; Tremblay and Dick, 2016). By virtue of this evidence, a distinction has been made between a basic, or core language circuit, which is surrounded by a network of supporting areas (Fedorenko, 2014).

### An updated model of the language regions

The current understanding of the basic speech circuit fits closely the organization of auditory networks in the monkey, including as a major component the direct connection between Broca's and Wernicke's areas via the AF (Figure 2B). This tract connects bidirectionally the core of Broca's area (Brodmann's areas 44 and 45), and the ventral premotor cortex according to some authors (Friederici, 2011), with regions of the posterior superior temporal lobe, including the ventral posterior STG, the posterior STS and part of the MTG (Rilling et al., 2008; Petrides, 2014; Figure 2B). The above mentioned area Tpt partly fits the termination of the AF, and has been considered by some as the core of Wernicke's region (Galaburda and Sanides, 1980). A related area is Spt, which is defined by functional activations during verbal working memory tasks. Since Tpt is defined cytoarchitectonically, and Spt is defined functionally, the relation between both regions is not yet clear, although they have been proposed to overlap (Hickok et al., 2003).

Beside the AF, there is a profuse connection between the ventrolateral prefrontal and premotor cortices on one side, and the inferior parietal lobe on the other, via the ventral SLF (Aboitiz and García, 1997; Petrides, 2014). This tract is termed by other authors as the anterior segment of the AF (Catani and Bambini, 2014). The inferior parietal lobe, also called Geschwind's area, is a multimodal region in which sensory modalities converge, and where mechanisms of motor program selection take place (Catani et al., 2005). Furthermore, the posterior segment of the middle longitudinal fasciculus (MLF, also termed the posterior segment of the AF) connects posterior auditory areas with the inferior parietal lobe, thus making up a triangular network with Broca's area, Wernicke's area and the inferior parietal lobe (Geschwind's area) at the respective vertices (Aboitiz and García, 1997; Catani et al., 2005; Catani and Bambini, 2014; Petrides, 2014; see Figure 2A). This circuit, together with the AF, has been dubbed the dorsal pathway, and is involved in sequential and structural analyses of phonology and grammar (at least complex, embedded grammatical forms). In addition to this projection, recent studies have unveiled a ventral language pathway, running along the superior temporal lobe and reaching the ventrolateral prefrontal cortex (specifically, areas 45 and 47) through the anterior temporal pole and the extreme capsule. This projection has been related to lexical and semantic linguistic processing (Saur et al., 2008; Catani and Bambini, 2014; Petrides, 2014), although other studies indicate involvement of the dorsal pathway in these functions as well (Rilling et al., 2012).

Analyses of resting state functional connectivity have shown that posterior Broca's area (area 44) correlates in activity with the posterior auditory cortex and anterior inferior parietal lobe, presumably via the AF and ventral SLF, and has said is considered to be involved in phonological and complex syntactic processing. This can be referred to as part of an auditory-vocal articulatory network, that is directly linked with premotor and motor regions controlling vocal and orofacial musculature (Petrides, 2014). On the other hand, anterior Broca's region (area 45) is functionally embedded in a multimodal network involving the posterior inferior parietal cortex via the dorsal pathway (AF/SLF), and the anterior temporal lobe and STS via the ventral pathway, which interfaces with visual networks involved in stimulus identification and action processing (Binder and Desai, 2011; Friederici, 2011; Nelissen et al., 2011; Petrides, 2014; Beauchamp, 2015). This poses area 45 as a critical node linking the articulatory network with surrounding multimodal networks conveying lexico-semantic and syntactic information (Petrides, 2014).

Other brain systems involved in speech and language are subcortical nuclei like the cerebellum, basal ganglia, hippocampus and thalamus, which have extensive connections with the language-related cerebral cortex. Particularly, the cerebellum has closely coevolved with the cerebral cortex in mammals and primates (Herculano-Houzel, 2010), and there is growing evidence that it contributes not only to sensorimotor coordination of speech and sign language, but also to higher cognitive functions, participating in tasks requiring verbal working memory, verbal fluency and in general, phonological and semantic processing (Vias and Dick, 2017). Further research is strongly needed to unveil the specific participation of these structures in speech and language (see Aboitiz, 2017).

### Lateralization of speech

Although the left cerebral hemisphere is commonly said to be dominant for language, recent evidence has shown that speech perception and production are bilateral processes, with the right hemisphere specializing in low frequency syllabic sampling of the stimulus, and the left hemisphere specializing in high frequency phonemic processing (Hickok and Poeppel, 2007; Poeppel, 2014). Furthermore, prosody and music (in musically non-trained individuals) is better represented in the right hemisphere, and depends on both the dorsal and ventral pathways, where the dorsal pathway conveys categorization and motor control, and the ventral pathway is dedicated to sound analysis (Sammler et al., 2015). Prosody and syntax are highly tuned, which is relevant for making inflections and punctuating speech. The corpus callosum is needed for this synchronization, as revealed by the absence of a N-400-like evoked potential termed ELAN, that marks syntactic-prosodic incongruencies, in patients con lesions in the posterior but not the anterior corpus callosum, implicating parieto temporal areas in this interaction (Sammler et al., 2010).

Anatomically, the Sylvian fissure has different shapes in both hemispheres, being horizontal in the left hemisphere, and curving upwards to the parietal lobe in the right hemisphere (Aboitiz et al., 1992). Furthermore, the AF has been reported to be more robust in the left than in the right hemisphere since birth (Perani et al., 2011), while the ventral branch of the SLF shows the reverse asymmetry, being amplified in the right hemisphere (Budisavljevic et al., 2015). Whether the gross anatomical and tractographic asymmetries correlate with each other remains to be established. A recent study combining tractography and functional connectivity in a semantic decision task, found that the left AF is more robustly connected with the lateral temporal cortex in the left hemisphere, but with the inferior parietal lobe in the right hemisphere (Takaya et al., 2015). Nonetheless, a recent review indicates that there are some inconsistencies across studies when determining the asymmetry of the AF (Wilkinson et al., 2017).

### From auditory-vocal to speech networks

As shown above, humans and monkeys display largely similar networks of auditory-prefrontal connectivity, indicating that the speech circuit emerged in evolution from a template existing in the last common ancestor. However, tractographic analyses revealed a significant difference in the development of the AF and ventral SLF, which are more robust, compared to the ventral pathway, in humans than in macaques (Figure 2; Aboitiz and García, 1997; Aboitiz et al., 2006a, 2010; Rilling et al., 2008, 2012; Aboitiz, 2012; Catani and Bambini, 2014; Petrides, 2014; Rilling, 2014). Nonetheless, tractographic evidence lacks the resolution of animal hodological techniques, and the separation of the AF from neighboring tracts can be problematic, especially as white matter becomes increasingly complex in larger brains (Petrides, 2014). The projection from the superior temporal lobe (Wernicke's region in the human) to the inferior parietal lobe (Geschwind's region) has been claimed to have strengthened in human evolution as well (Aboitiz and García, 1997; Aboitiz et al., 2006a; Catani and Bambini, 2014). Complying with these findings, the connectivity of the laryngeal motor cortex with inferior parietal areas was found to be as much as seven fold stronger in the human than in the macaque (Kumar et al., 2016). This projection may be indirectly connected with auditory projections to inferior parietal areas (Hickok, 2017). The strengthening of direct or indirect auditory-frontal connectivity via the dorsal pathway may have been achieved in more than one way. One is increasing the number of fibers connecting the respective regions, and a second one is changing the fiber composition and the tract integrity of the AF and related tracts, yielding enhanced functional connectivity. In this line, imaging analyses have revealed a weaker resting state functional connectivity between auditory and ventral prefrontal regions in the macaque than in the human (Mantini et al., 2011; Neubert et al., 2014; Petrides, 2014).

Nonetheless, comparative tractographic evidence suggests that the expansion of the dorsal pathway including the AF may have been gradual in primate evolution, as in the chimpanzee this component displays an amplification that is intermediate between the human and the monkey (Rilling et al., 2008). What functions does the chimpanzee AF subserve are an intriguing mystery, as like monkeys, apes are supposed to have limited vocal learning capacity. One possibility is that the AF of the chimpanzee participates in lip-sound associations, or more generally, orofacial control and its association to sound. Furthermore, while both the chimpanzee and the human share a projection between the auditory STG and the ventrolateral prefrontal cortex, only in humans there is a robust projection from the dorsal pathway, that ends in the multimodal STS and MTG (Rilling et al., 2008). There is discussion as to whether this component is part of the monosynaptic AF or whether it corresponds to fibers from the posterior MLF (Petrides, 2014; see Figure 2). Petrides (2014) also argues that the expansion of the temporoparietal junction of the human brain relative to other apes (Margulies et al., 2016), may have produced a ventral displacement of areas located more dorsally in other primates, concomitant to a lengthening of the AF into the MTG. In this context, an interesting test would be to study the anatomy of the AF in microcephalic brains, who despite their small brain sizes, some still have linguistic abilities beyond those of language-trains chimpanzees. In any case, this descending component of the tract is undoubtedly part of the dorsal pathway that conveys multimodal information and may be involved in lexicosemantic and possibly grammatical processing (Rilling et al., 2008).

Non-human primates and especially chimpanzees, show brain asymmetry at the behavioral (for example, hand dominance), and gross anatomical and tractographic levels (specifically, they have a leftwardly asymmetrical AF) in auditory-vocal areas (Rilling et al., 2012). Nonetheless, functional and behavioral asymmetries are much more pronounced in humans than in other primates, and this might partly explain the consolidation of the speech circuit in the left hemisphere of most humans.

## The phonological loop

Alan Baddeley (Baddeley and Hitch, 1974; Baddeley, 2007) proposed a model of working memory as a transient, limited capacity memory system that keeps information online, to be used in the near future. One of the components of this system is the phonological loop, a system involved in the transient maintenance of phonological sequences while performing a task. More than residing in a specific cortical region, the storage of phonological items in memory seems to depend on the sustained activation of a sensorimotor circuit encompassing posterior auditory areas (particularly, area Spt mentioned above) and the ventrolateral prefrontal cortex, in which the dorsal pathway may be a key element (Hickok, 2017). This mechanism is supported by inferior parietal regions that contribute attentional resources and select motor articulatory programs that transiently stabilize the phonological trace (Aboitiz, 2012, 2017; Rauschecker, 2012; Fedorenko, 2014).

### A key innovation

Baddeley considered that the phonological loop did not evolve so much to process language, but rather to increase language learning capacity, and showed that verbal working memory in children is associated with their subsequent vocabulary acquisition (Baddeley, 2007). In this line, we have developed the hypothesis that the phonological loop is a character uniquely human among primates, that was crucial for the acquisition of speech in our species' early evolution. This process was accompanied by the development of auditory-vocal circuitry involving the AF and other components of the dorsal pathway, together with the increasing descending control over vocal cranial motor nuclei (Aboitiz and García, 1997; Aboitiz et al., 2006a, 2010; Aboitiz, 2012, 2017; see also Catani and Bambini, 2014).

Supporting this proposal, there is evidence that points to an increased auditory-vocal anatomical and functional connectivity via the dorsal pathway in humans compared to monkeys (see above), and behavioral experiments have shown that as opposed to visual memory, monkeys are strongly limited in auditory long and short term memory (Scott et al., 2012; Scott and Mishkin, 2016). Furthermore, tractographic integrity of the AF has been associated with verbal working memory, verbal fluency and sentence comprehension in humans, and its development in childhood correlates with increasing language abilities (Yeatman et al., 2011; Skeide et al., 2016; Schomers et al., 2017). Certainly, other mechanisms beside working memory capacity were involved in the origin of speech at its different levels, but the argument is that the phonological loop facilitated these acquisitions.

### The phonological loop amplified

Verbal working memory is not unitary, and operates at very different levels, phonological, syntactic, lexical, and semantic (Caplan and Waters, 1999). These levels depend on different but highly interacting neural networks, as for example phonological working memory relies on the dorsal pathway and the AF (Schomers et al., 2017), while lexicosemantic working memory depends more, but not exclusively, on the ventral pathway, in compliance with the organization of the auditory system (Binder and Desai, 2011). Syntactic working memory, especially complex, embedded grammatical forms, has been proposed to depend on the dorsal pathway (Friederici, 2011; Goucha et al., 2017). Nonetheless, some syntactic processes have been found to depend on the ventral pathway, especially when involving interpretation of meaningful discourse (Griffiths et al., 2013).

How did this complex set of networks evolve? I will propose here a sequence of five overlapping stages in the evolution and amplification of the auditory-vocal circuitry in the human lineage. Firstly, like other primates, early australopithecines possibly relied more intensely on the ventral auditory pathway to process vocalizations and associating them to visual stimuli representing faces and gestures in the anterior ventrolateral prefrontal cortex (Romanski, 2007). Secondly, a main innovation was the increased neural control of vocalizations and orofacial movements via the laryngeal and orofacial motor cortex, directly connected both to brainstem motor nuclei and inferior parietal areas (Kumar et al., 2016; Hickok, 2017). Thirdly, atop of this basic circuit, the activation of an auditory-vocal reciprocal loop, relying on a bidirectional connection between Broca's region with posterior auditory areas via the AF and ventral SLF, enabled the learning of complex vocal utterances by imitation, establishing the basic components of the phonological loop and enhancing auditory-vocal working memory capacity (Petrides, 2014). For example, in a phonological working memory task using multisyllabic pseudowords, the areas activated during maintenance of the stimulus on mind were posterior temporal area Spt (see above) and the nearby posterior STS, where the integration of phonemes into word forms takes place. While the posterior STS has been related to the AF (Petrides, 2014), the connectivity of area Spt remains to be determined. This circuit is the core network for vocal articulation, and its functional amplification is probably a key development in the human lineage, allowing early humans to learn increasingly complex phonological, or pre-phonological sequences. This may have been used initially for social bonding, but perhaps also for transmitting simple information about events or objects, as in vervet monkey alarm calls that signal specific predators. The structure of vervet alarm calls is largely innate, but their referentials or “meanings” are dependent on social experience (Seyfarth et al., 1980). In early humans, these vocal calls may have become learned by virtue of increasing vocal plasticity. As the vocal messages became increasingly complex, more extended cortical regions became recruited, particularly inferior parietal regions projecting to Broca's area, that also provided a rudimentary order to the sequences of vocalizations, possibly relying on constraints associated with sensorimotor programming.

In a fourth event, the ventral auditory pathway, processing the sound characteristics of vocalizations, strengthened associations with the ventral visual pathway via the STS, where information about objects, events and actions is processed (García et al., 2014). In addition, the development of a dorsal pathway projection to the MTG in humans but not in apes may have contributed to transmit lexical-semantic information and possibly some elements of syntax into the dorsal pathway (Rilling et al., 2008). The auditory ventral pathway is heavily connected with anterior Broca's area and neighboring regions (areas 45 and 47), which integrates articulatory information from the dorsal stream with auditory-lexical inputs from the ventral stream, facilitating the transformation of phonological representations into vocal motor programs (Skeide and Friederici, 2016). As associations between learned vocalizations and visual representations, originating along the STS, became conventionalized by cultural or proto-cultural development, a primitive lexicon appeared, providing meaning to the phonological sequences and slowly forming a proto-lexicon (García et al., 2014). This early, proto-lexical stage may have lasted for a long time, while modern speech and grammar are probably more recent acquisitions (Bickerton, 2014). For reasons of space, it is impossible to discuss the emergence of grammar in this article, but I have argued elsewhere that syntactic rules appeared to translate complex visuomotor representations of actions and events into hierarchical phonological structures and vice versa (Aboitiz et al., 2006b; Aboitiz, 2017, in press). This perspective differs from the canonical view of grammar as an encapsulated device, separate from other cognitive systems (Hauser et al., 2002).

## Discussion: a brief scenario of speech origins

This review has provided comparative anatomical, behavioral, and functional evidence that in my view points to a continuous evolution of the vocal system and its neural control, from non-human primate vocalizations to at least the early stages of human speech. On the other hand, exponents of the mirror system hypothesis tend to disregard the role of non-human primate vocalizations, and especially downplay the emergence of prosody in the origin of speech. What comes below is a tentative scenario of early human evolution, in which speech evolved as a response to selective forces that resulted in both biological and cultural adaptations to yield modern language.

Australopithecines originated some 4 million years ago, and underwent a quite different evolutionary trajectory than that of their ancestors and sister taxa. These were successful bipedal species, with an ecology and social organization probably similar to that of macaques living in open spaces (Meindl et al., 2018). Australopithecine descendants, belonging to the genus *Homo*, probably developed a quite intense social life compared to other primates, concomitant with increased levels of prosocial neurotransmitters in the subcortical basal ganglia (Raghanti et al., 2018). In addition, early humans developed a culture in which tool making and fire control became essential elements (although these may have started already in Australopithecines), mainly due to a highly sophisticated digital dexterity, possibly far beyond that found in other primates. In addition to this, I propose that Australopithecines and early *Homo* communicated intensely with vocal signals. Darwin already proposed that initially, vocal communication was more similar to music than to speech, which has been updated as the “musical protolanguage,” or prosodic hypothesis (Fitch, 2010; Hickok, 2017). Early humans probably engaged in turn-taking conversations that may have lasted for a long time and served to strengthen bonds, especially between mother and child, but also to communicate emotional states, as seen in marmoset monkeys (Takahashi et al., 2013, 2016). Other non-primate examples are highly social mammals like cetaceans, who use learned vocalizations to promote social bonds and group coordination (King and McGregor, 2016). Each individual dolphin in a group has its own specific vocalization that has been learned from early life (King et al., 2016). Cetaceans, similarly to elephants, orangutans and other highly social mammals, have been shown to be able to imitate the human voice (Ridgway et al., 2012; Stoeger et al., 2012; Lameira et al., 2016; Abramson et al., 2018). Supporting this perspective, increasing vocal complexity has been associated with more elaborate social behavior in birds, where cooperative breeding correlates with vocal richness. This is consistent with the idea that social complexity by itself may be a selective force driving vocal evolution (Leighton, 2017). Australopithecines had brains not much larger than those of chimpanzees, and the expansion of human brain size does not take place until later. Yet, acquisition of vocal plasticity does not require a large brain, as can be shown by the example of echolocating bats, who are highly social and good vocal learners (Morell, 2014). Probably, brain size increased concomitant with the progressive development of linguistic and social skills, as there was increasing cognitive pressure with the more complex communication and social life that was emerging (Aboitiz, 2017).

Nonetheless, early human communication was probably multimodal, using both vocalizations and gestures, as it is today. The vocal learning skills of early humans may have been put to use to mimic the sounds of animals, water, the wind, or other elements nearby, together with gestural pantomime (García et al., 2014). Likewise, they may have developed learned alarm calls that signal specific predators, that were accompanied by gesticulations (Seyfarth et al., 1980). This emerged into a primitive, gestural-vocal proto-semantic system (García et al., 2014). However, pantomimes and manual gestures probably never went much beyond the stage observed in normally speaking modern humans. On the other hand, the elaboration of auditory-vocal networks and the gradual consolidation of the phonological loop eventually enabled our ancestors to start communicating increasingly complex meanings through the voice (García et al., 2014; Aboitiz, 2017). In later stages, the acquisition of semantics and a primitive lexicon may have been essential for the separation between both kinds of expression, and possibly contributed to the lateralization of these functions, with phonology and speech on the left hemisphere and music/prosody in the right hemisphere, both communicating via the corpus callosum (Sammler et al., 2015).

For these events to occur, a tight control of lips, tongue and the vocal folds must have taken place. Furthermore, a precise coordination between the vocal folds and the upper vocal tract may have evolved in these species, to synchronize vocalizations with mouth movements, as is seen in gelada baboons (Ghazanfar and Takahashi, 2014a). The development of direct cortical control of these brainstem nuclei was most likely not a difficult evolutionary step, that could have been achieved with minimal genetic changes (Gu et al., 2017), and may have also developed together with increasing cortical size (Herculano-Houzel et al., 2016). For our ancestors and not for other primates, there was a strong selective benefit in developing vocal learning capacity, possibly in the context of an increasingly complex social organization.

Summarizing, it was intense sociality, together with a tool-making culture and specific ecological circumstances, that selected for more complex vocalization and gestural capacity, generating a virtuous cycle that eventually exploded as a functional phonological loop gradually consolidated in our recent ancestors. Furthermore, human brain size increased in response to pressure for increasing communication and technological abilities, where larger brains enabled more complex communication and behavioral innovations, generating further communicative and cognitive pressures (Bickerton, 2014; Aboitiz, 2017). This virtuous cycle may have had an exponential dynamics, being quite slow for a long time, until a threshold was reached that launched human behavior into modern language. While we will probably never know exactly which circumstances led to the acquisition of speech nor when it happened, this article has aimed to show evidence for strong homology between the auditory-vocal neural circuitry in humans and non-human primates.

## Author contributions

The author confirms being the sole contributor of this work and approved it for publication.

### Conflict of interest statement

The author declares that the research was conducted in the absence of any commercial or financial relationships that could be construed as a potential conflict of interest.

## Abbreviations

A: primary auditory area
AC: anterior cingulate cortex
AF: arcuate fasciculus
AM: amygdala
DLF: dorsolateral frontal cortex
EC: extreme capsule
ILF: inferior, longitudinal fasciculus
LC: laryngeal and orofacial cortex
MLF: middle longitudinal fasciculus
MTG: middle temporal gyrus
NA: nucleus ambiguous
PAG: periaqueductal gray
SLF: superior longitudinal fasciculus (ventral)
STG: superior temporal gyrus
STS: superior temporal sulcus
UF: uncinate fasciculus
V1: primary visual area.
